# Worsening of Angina Following Nitroglycerin Administration: A Case Report of the Interplay With Undiagnosed Myocardial Bridge

**DOI:** 10.7759/cureus.40091

**Published:** 2023-06-07

**Authors:** Liliana P Guevara-Bermudez, Olga Toleva

**Affiliations:** 1 Department of Hospital Medicine, Emory Saint Joseph's Hospital, Atlanta, USA; 2 Department of Medicine, Division of Hospital Medicine, Emory University School of Medicine, Atlanta, USA; 3 Department of Medicine, Division of Cardiology, Emory University School of Medicine, Atlanta, USA; 4 Emory Women’s Heart Center, Emory Saint Joseph’s Hospital, Atlanta, USA

**Keywords:** chest pain syndrome, coronary artery disease, nitroglycerin (ntg), coronary vasospasm, left heart catheterization, functional left heart catheterization, ischemia with open coronary arteries, myocardial bridge

## Abstract

Myocardial bridge (MB) is a congenital abnormality where part of a coronary epicardial artery runs under the myocardium fibers and is compressed in systole; this becomes more pronounced when nitroglycerin (NTG) is administered. In this report, we describe the case of a 40-year-old African American man who presented with chest pain that did not respond to NTG or isosorbide mononitrate and was only partially relieved by narcotics. His past medical history was significant for coronary artery disease (CAD) with a stent into the left anterior descending artery (LAD) several months prior, hypertension, hyperlipidemia, paroxysmal atrial fibrillation, sick sinus syndrome, permanent pacemaker, pulmonary embolism, and cerebral vascular accident. No explanation for his angina was found either in the previous outpatient left heart catheterization (LHC) procedures demonstrating LAD stent patency or initial chest pain workup upon admission. Functional LHC procedure with adenosine infusion and acetylcholine provocation demonstrated endothelial dysfunction with notable epicardial spasm and MB of the LAD that worsened with NTG. Cardiology advised dual antiplatelet therapy and a statin as part of treatment for CAD and a calcium channel blocker with a bradycardic effect (e.g., diltiazem, verapamil) for the MB and coronary vasospasm, and avoidance of NTG and long-acting nitrates (e.g., isosorbide mononitrate), which can cause reflex tachycardia and worsen angina from MB. A selective serotonin reuptake inhibitor was added for increased cardiac nociception. The patient’s pain resolved, and he was discharged. MB is an important alternate etiology to consider when chest pain does not respond to NTG administration for adjustment of treatment modalities. The initial treatment for this patient’s pain with NTG likely exacerbated symptoms by reducing intrinsic coronary wall tension and subsequently increasing reflex sympathetic stimulation of contractility of the left ventricular myocardium, which can, in turn, increase anginal symptoms and ischemia.

## Introduction

Myocardial bridge (MB) is a congenital cardiac structural abnormality where a small area of an epicardial artery, most frequently the left anterior descending artery (LAD) [1], runs under the coronary muscle fibers and is compressed in systole. This becomes more pronounced when nitroglycerin (NTG) is administered. While the reasons for this are not fully understood, researchers have hypothesized that NTG may exacerbate systolic compression of the cardiac bridge via vasodilation of the surrounding segments [1], or potentially its effect on vessel wall compliance, as well as increased sympathetic contractility [2].

The prevalence of MB is estimated to be 19% [3], with ranges between 6% and 42% described in autopsy studies, depending on the method used [4]. Most cases are asymptomatic [5]. There are varying degrees of MB in both length and depth [6], with corresponding impacts on cardiac function and pain [7,8]. 

This case highlights the need to investigate alternate etiologies including MB when chest pain does not respond to NTG administration, and to adjust treatment modalities accordingly.

A vignette of this case was previously highlighted as a Case of the Month in the Emory Daily Pulse online blog in September 2022, and was presented orally at the Emory Department of Medicine Research Day in Atlanta, Georgia, United States, on October 27, 2022.

## Case presentation

A 40-year-old African American man presented with retrosternal chest pain that did not respond to NTG and isosorbide mononitrate and was only relieved by narcotics. He had a previous percutaneous coronary intervention with a stent into the LAD several months prior for obstructive coronary artery disease (CAD). He also had a history of hypertension, hyperlipidemia, paroxysmal atrial fibrillation, sick sinus syndrome, permanent pacemaker, pulmonary embolism, and cerebral vascular accident. He endorsed recurrent chest pain episodes after initial stent implantation, necessitating repeated left heart catheterization (LHC) on at least two occasions as an outpatient. Each time, the LAD stent was found to be patent, and no clear explanation for his angina symptoms was identified.

As noted above, the patient’s initial LHC procedure demonstrated a patent stent and open coronaries. During the current presentation, he was admitted to the hospital and had an appropriate workup for chest pain, including negative troponins, EKG with non-ischemic changes, and a computed tomography (CT) scan of his chest with no evidence of pulmonary embolism.

As his angina did not resolve with NTG, the consulting cardiologist suggested functional LHC to assess coronary physiology, microvascular function, and epicardial vasospasm using intracoronary (IC) acetylcholine (ACH) provocation testing.

**Figure 1:**
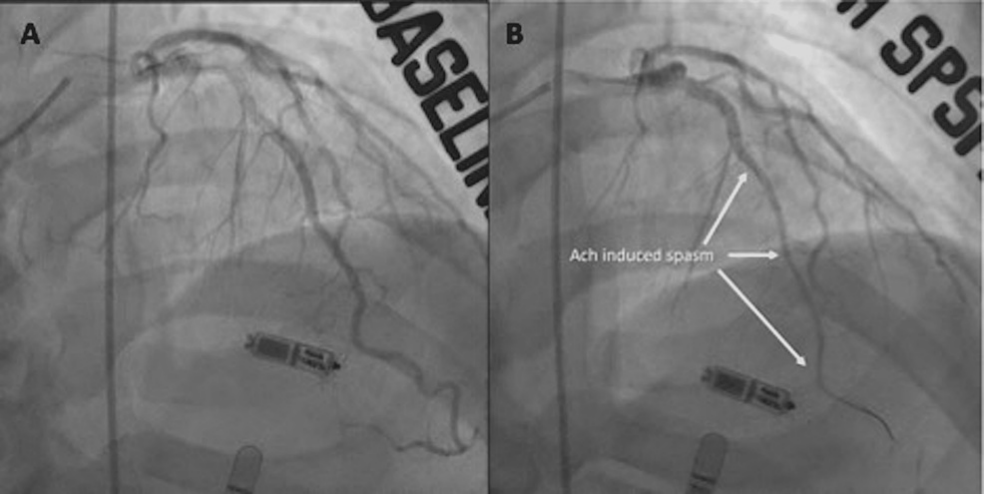
Notable epicardial spasm of LAD following acetylcholine provocation in the myocardial bridge segment A. Baseline angiogram of the LAD; B. Angiogram of the LAD in the myocardial bridge segment with spasm post acetylcholine provocation LAD: left anterior descending artery; Ach: acetylcholine

**Figure 2:**
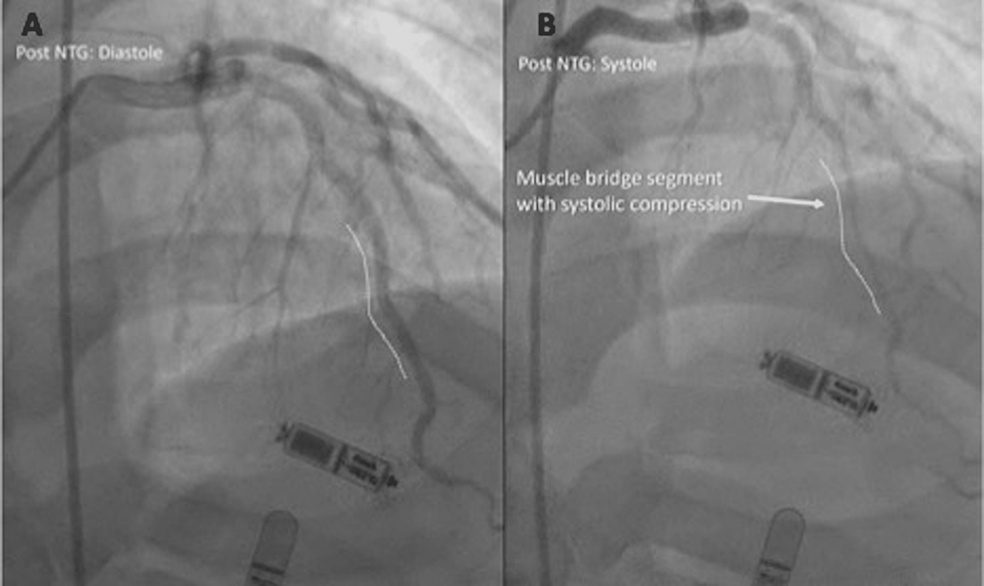
LAD appearance post nitroglycerin A. LAD in diastole: no compression from the myocardial bridge; B. LAD in systole: significant compression from the myocardial bridge LAD: left anterior descending artery;; NTG: nitroglycerin

During the functional LHC procedure, we used bolus thermodilution pressure wire (CoroFlow Cardiovascular System, Coroventis Research AB, Uppsala, Sweden; PressureWire™ X Guidewire, Abbott, Abbott Park, Illinois, United States). We utilized a spasm dose of ACH 100 mcg administered in the LAD via a microcatheter infused over three minutes. Severe spasm response was observed based on angiographic constriction >90% of the mid to distal LAD vessel as compared to baseline. When we measured the vessel diameter by quantitative coronary angiography at baseline, it was 1.85 mm but decreased to 0.4 mm during spasm provocation. There was associated severe chest pain reproducing the symptoms and electrocardiographic changes, ST-segment elevation in leads V1-6, 1, and aVL, confirming the diagnosis of coronary vasospasm. Following this, IC NTG was administered, which reversed the epicardial spasm but made the MB segment more pronounced as seen on the angiogram (Figures 1-2).

The cardiology consultant advised dual antiplatelet therapy and a statin as part of treatment for CAD and endothelial dysfunction, a calcium channel blocker with a bradycardic effect (e.g., diltiazem, verapamil) for coronary vasospasm or beta blocker to reduce systolic compression on the MB, and avoidance of NTG and long-acting nitrates (e.g., isosorbide mononitrate) to reduce vasodilatory reflex tachycardia, which can worsen the muscle bridge.

The patient was started on diltiazem and sertraline, continued clopidogrel, apixaban, and atorvastatin, and was instructed to avoid NTG and isosorbide mononitrate, as well as narcotics in the future. He was discharged with the resolution of his chest pain.

## Discussion

The initial treatment for this patient’s pain with NTG was ineffective; instead, it acutely worsened his pain, probably due to vasodilation and reflex tachycardia that worsened the effect of the muscle bridge with shorter diastole and more ischemia. However, the exact mechanism is not entirely known. NTG likely exacerbated his symptoms by reducing intrinsic coronary wall tension and subsequently increasing reflex sympathetic stimulation of contractility of the left ventricular myocardium, which can, in turn, increase anginal symptoms and ischemia [2].

Chest pain is extremely common in the hospital setting, frequently results from CAD, and is usually treated successfully with NTG. However, in cases of MB or recurrent unexplained chest pain episodes, providers should investigate further, as the management of uncommon etiologies can be significantly different from normal courses of treatment [5,9,10]. Specifically, NTG is not appropriate for every case of chest pain in the setting of CAD, and in some cases can worsen ischemia; in the case of MB, NTG can accentuate systolic compression of bridged segments [1,11,12].

Several case reports have described patients presenting with angina resulting from MB [13-17]. Other pain management strategies should be pursued and narcotics should be avoided when NTG fails to relieve chest pain, as they may mask symptoms, delay appropriate diagnosis, and create dependency without having a direct effect on the condition. Functional LHC should be considered in patients with open coronaries and anginal symptoms, as they may reveal endothelial dysfunction, epicardial spasm, or a functionally significant MB, as in this case.

The presence of muscle bridge can be identified non-invasively by coronary CT angiography; however, the hemodynamic effect of the muscle bridge and the superimposed vasospasm can only be seen in invasive LHC with additional modalities like intravascular ultrasound, dobutamine provocation, or ACH spasm provocation in the catheterization laboratory.

In this case, once MB and superimposed spasm were documented invasively, future refractory angina presentations can be evaluated non-invasively with a nuclear stress test, knowing that the spasm and MB will not cause ischemia on such a test, and if there is new ischemia, it is most likely due to new or progression of existent CAD or in-stent restenosis.

## Conclusions

The chest pain syndrome in the presence of MB can be challenging to treat especially when coronary vasospasm coincides. Administering NTG can worsen the effect of MB on myocardial ischemia and therefore the use of non-dihydropyridine calcium channel blockers is the preferred option due to the beneficial bradycardic effect for the MB and vascular smooth muscle cell relaxing effect on coronary vasospasm. Using NTG for chest pain in patients with CAD is usually the first action of healthcare providers; however, not every case of chest pain requires NTG as a long-term treatment. When NTG is not effective or increases chest pain, providers should investigate alternate etiologies.
